# Releasing stored lipids to fuel migration and reproduction in the eel, *Anguilla australis*—a role for 11-ketotestosterone?

**DOI:** 10.1007/s10695-025-01480-4

**Published:** 2025-04-07

**Authors:** P. Mark Lokman, Deborah Lynch, Peter S. Davie, Erin L. Damsteegt

**Affiliations:** 1 Department of Zoology, University of Otago, Dunedin 9054, PO Box 56, Aotearoa, New Zealand; 2 Institute of Veterinary, Animal and Biomedical Sciences, Massey University, Private Bag 11-222, Palmerston North 4442, Aotearoa, New Zealand

**Keywords:** *Anguilla*, Migration, 11-ketotestosterone, Lipolysis, Gene expression, White muscle

## Abstract

Migrating freshwater eels depend on the mobilisation of stored lipids to successfully arrive at their distant spawning locations. As 11-ketotestosterone (11KT) can increase the lipid-transporting capability and enhance gonadal lipid uptake in eel, we hypothesized that this androgen would also regulate lipid mobilisation from its stores. To address this hypothesis, we first sampled residential (yellow) and migrating (silver) short-finned eels from the wild and evaluated the expression of 24 genes encoding lipolytic or lipogenic enzymes, as well as those encoding both nuclear androgen receptors, by NanoString analysis. Plasma 11KT levels in silver eels were dramatically increased, and mRNA levels of more than half of all target genes were higher in silver eel muscle; none of the target genes was significantly downregulated. Gene expression profiles in white muscle from wild-caught eels were subsequently compared with those from yellow and silver eels subjected to implantation with sustained-release implants containing 11KT. Several weeks of exposure resulted in plasma levels of 11KT that resembled those of wild-caught eels and resulted in a dose-dependent increase in gonadosomatic and hepatosomatic index; however, target gene expression profiles in muscle were barely affected. We conclude that lipid physiology in white muscle of silver eels is notably different from that in yellow eels, and that 11KT is not responsible for the differentially expressed gene profile between yellow and silver short-finned eels.

## Introduction

Before embarking on long-distance migrations to far-away spawning grounds, anguillid eels undergo what is sometimes considered to be a second metamorphosis, referred to as silvering. This event sees immature ‘yellow’ eels, that have taken-up residence in freshwater bodies, transform into pre-pubertal / early maturing ‘silver’ eels that are pre-adapted to oceanic conditions. Numerous morphological, behavioural and physiological changes occur, including, but not limited to, enlargements of key organs such as heart, liver, eyes and gonad, a shift in function of digestive tract away from nutrient absorption and towards osmoregulation, and a darkening of the fins and integument (Lokman et al. 1998, 2003; Aoyama and Miller 2003; Tsukamoto 2006). Many of the changes associated with silvering can be induced by exogenous treatment with 11-ketotestosterone (11KT) (Rohr et al. 2001; Setiawan et al. 2012; Sudo et al. 2012; Hagihara and Sudo 2023), an androgen that naturally increases in concentration during silvering (Lokman et al. 1998).

We previously reported that 11KT has notable effects on lipid physiology in a short-finned eel, *Anguilla australis* (Damsteegt et al. 2016). These effects are manifested as increased expression of genes encoding apolipoprotein B (Apo B), Apo E and microsomal triacylglyceride transfer protein, which we considered to reflect increased lipid packaging and vascular transportation capability (Damsteegt et al. 2016). At the same time, 11KT strongly affects lipid accumulation into developing oocytes, both in vivo and in vitro (Lokman et al. 2007; Divers et al. 2010; Endo et al. 2011; Damsteegt et al. 2015a), thus supporting the nutritional needs of the future embryo. Since the bulk of oocyte growth occurs once eels fast and leave continental waters *en route* to the spawning grounds, a well-coordinated approach to freeing up lipids from storage depots is key. Given the effects of 11KT on lipid packaging, vascular transport and ovarian accumulation, we posit that this androgen also stimulates lipid mobilisation from peripheral deposits; accordingly, a single hormonal mediator would act as a master co-ordinator of lipid physiology during migration and oogenesis.

In eel, fats are stored in liver, muscle, subcutaneous sites, in the ovary, and around the viscera, white muscle being considered the main store for lipids in the form of triglycerides (c.f., Svedäng and Wickström 1997; Van Ginneken et al. 2018; Parzanini et al. 2021). Storage of fats in white muscle is associated with adipocytes and although red muscle typically contains a higher lipid content than white muscle (see: Sheridan 1994), the much larger contribution of white muscle to total muscle mass (Pankhurst 1982) reinforces its importance as a major depot in the eel. To test the hypothesis that androgens contribute to mobilisation of triglycerides from white muscle storage sites, we first compared the expression of lipid physiology-related target genes in yellow and silver short-finned eels (‘Field survey’ for baseline data) and then evaluated the effects of slow-release 11KT-implants on white muscle gene expression profiles in female short-finned eels in yellow and silver stages. Gene expression was quantified for 24 lipid physiology-related genes and both nuclear androgen receptor genes using the NanoString nCounter analysis (www.nanostring.com).

## Materials and methods

### Animals

Short-finned eels were caught in fyke nets from Lake Ellesmere, South Island, New Zealand, by professional eel fishermen during the austral autumn. Freshly caught eels were retrieved from nets set overnight; 12 females were selected, as much as possible on a size-matched basis, so as to obtain six residential immature ‘yellow’ and six migrating, early maturing ‘silver’ eels (for details, see Lokman et al. 1998). Fish were transferred into perforated 40 gallon plastic drums and placed in flow-through concrete tanks fed with spring water at 12 °C (c.f. Lokman et al. 1998) until sampling later that same day (see ‘Field survey’, in ‘Experimental design’).

Eels used for experimentation (see ‘Experiments’, in ‘Experimental design’) were maintained near the site of capture in concrete tanks with freshwater flow-through until shipment by road to laboratory facilities at the Department of Zoology, University of Otago, approximately 350 km south. Upon arrival to experimental facilities, fish were placed in recirculating 1000 L tanks containing ^1^/_3_ seawater at ambient indoor temperature until use. Fish were not fed throughout the acclimation or experimental periods. All experiments were approved by the Animal Ethics Committee, University of Otago, in keeping with ANZCCART guidelines (anzccart.org.nz).

### Experimental design

#### Field survey

Eels (six yellow eels, body weight BW = 836 ± 110 g; six silver eels, BW = 1076 ± 34 g) were euthanized in overdose benzocaine (0.3–0.4 g/L) and weighed, measured and drained of blood by removal of the tail. Blood was mixed with *ca* 1% (v/v) ethylenediaminetetraacetic acid (200 mg/mL) and centrifuged (10,000 g for 5 min) to obtain plasma for 11KT measurement (‘Radioimmunoassay for 11-ketotestosterone’). A portion of ovarian tissue was collected and placed in 1 mg/mL collagenase in eel ringer (Ijiri et al. 1995) to determine oocyte diameters (‘Determination of oocyte diameters’). Liver weight and ovary weight were recorded to determine hepato- and gonadosomatic indices (100 × organ weight/BW). A 2–3-cm thick ‘steak’ was collected, about 10 cm behind the anus (approximately ^1^/_3_ of the distance between anus and tip of the tail) and the skin, dermis and red muscle were carefully removed by scalpel and forceps. A small piece of the remaining dorsal white muscle was frozen on dry ice and stored at − 70 °C until use for lipid physiology-related gene expression analysis by NanoString Technologies (‘NanoString nCounter™ analysis’).

#### *Experiment I—*In vivo* 11-ketotestosterone dose response in female yellow short-finned eels*

Twenty-four yellow short-finned eels (BW = 684 ± 5 g) were acclimated to laboratory conditions for six days before being assigned randomly to one of four tanks. Fish in each tank were anaesthetized in 0.15 g/L benzocaine before being blood-sampled by syringe and subjected to treatment with an intraperitoneal implant of cholesterol/cellulose containing 11KT (0, 0.03, 0.1 or 0.3 mg/implant; animal groups are denoted by C, L0.03, M0.1, H0.3). The implants were prepared and administered as described earlier (Lokman et al. 2015). Fish were returned to their tanks and maintained at 15–17 °C in brackish water of 3–4 ppt salinity. Additional blood samples (< 0.3 mL) were collected on days 1, 4, 7 and 10 to evaluate the effects of the implants on plasma 11KT levels. After 3 weeks, eels received an overdose of benzocaine and were weighed, measured and dissected as described for the ‘Field survey’ above; however, only half of the white muscle samples (*n* = 3 fish/group) were randomly selected for the NanoString analysis.

#### *Experiment II—*In vivo* 11-ketotestosterone treatment of female silver short-finned eels*

Silver eels were starved for *ca* 7 months at 10–12 °C prior to experimentation to decrease endogenous levels of 11KT. Thereafter, water temperature was increased to 16 °C for 1 week prior to implantation of eels (*n* = 12; BW = 861 ± 31 g) with pellets that did, or did not, contain 0.3 mg 11KT. After 3 weeks, the experiment was terminated and fish were sampled as described above. In addition, a small piece of ovarian tissue was preserved in Baker’s calcium-formol for histological examination (‘Histology’).

### Radioimmunoassay for 11-ketotestosterone

Plasma levels of 11KT were determined from two technical replicates of each blood sample collected at euthanasia by validated RIA as reported previously (Lokman et al. 1998), using an 11KT antiserum that was gifted by Dr. David Kime (formerly University of Sheffield), as described in Kime and Manning (1982). All samples from a given experiment were run within a single assay, and inter-assay coefficients of variations (CV) were not, therefore, determined. The intra-assay CV was 10.2% and the minimum level of detection amounted to 0.13 ng/mL. No corrections were made for recovery of steroid (> 73%) during diethyl ether extractions.

### Determination of oocyte diameters

After digestion in collagenase for 2–3 h at room temperature and gentle inversion in the sample tube, the majority of eel ovarian follicles were readily freed from surrounding somatic tissue. Separated follicles were transferred to a glass 9-well spot plate and photographed with an Olympus SC100 camera connected to an Olympus BX51 compound microscope. All follicles in an image were counted, and the 20% largest-by-eye were measured using cellSens software. The measurements were sorted by size and the upper half (50% largest measurements) were retained for calculation of the average oocyte diameter for each individual. This approach allows for a sample size-independent estimate of oocyte size (see Lokman et al. 2007).

### Histology

Samples were processed, embedded into methacrylate resin (Technovit 7100, Kulzer GmbH, Germany) and prepared for histology according to manufacturer’s instructions and as outlined in Lokman et al. (1998). Sections were viewed with an Olympus BX51 microscope, digitally photographed using an Olympus SC100 camera and examined for the presence of follicular atresia.

### NanoString nCounter™ analysis

Total RNA was extracted using Trizol reagent (Life Technologies) according to manufacturer’s instructions. RNA quantity was assessed using Quant-iT™ RiboGreen™ RNA Assay Kit (Life Technologies) and the expression of target genes determined using the NanoString nCounter™ analysis, contracted to New Zealand Genomics Limited and run by Otago Genomics and Bioinformatics Facility. Prior to analysis, fluorescent probes were designed (‘CodeSet’, Table 1) and synthesized by NanoString for all target genes (24 enzymes, three reference genes and both nuclear androgen receptors), using sequence data from the National Center for Biotechnology Information and using unpublished in-house data from a *de novo* assembled eel ovarian transcriptome. A suite of lipase-encoding genes was chosen to ensure good representation of (intracellular) lipolytic activities, whereas synthase- and transferase-encoding genes were selected for their perceived lipogenic activities. Nuclear androgen receptors were included to allow for evaluation of androgen-binding activity. RNA was run as outlined in Bentley-Hewitt et al. (2016).

**Table 1:**
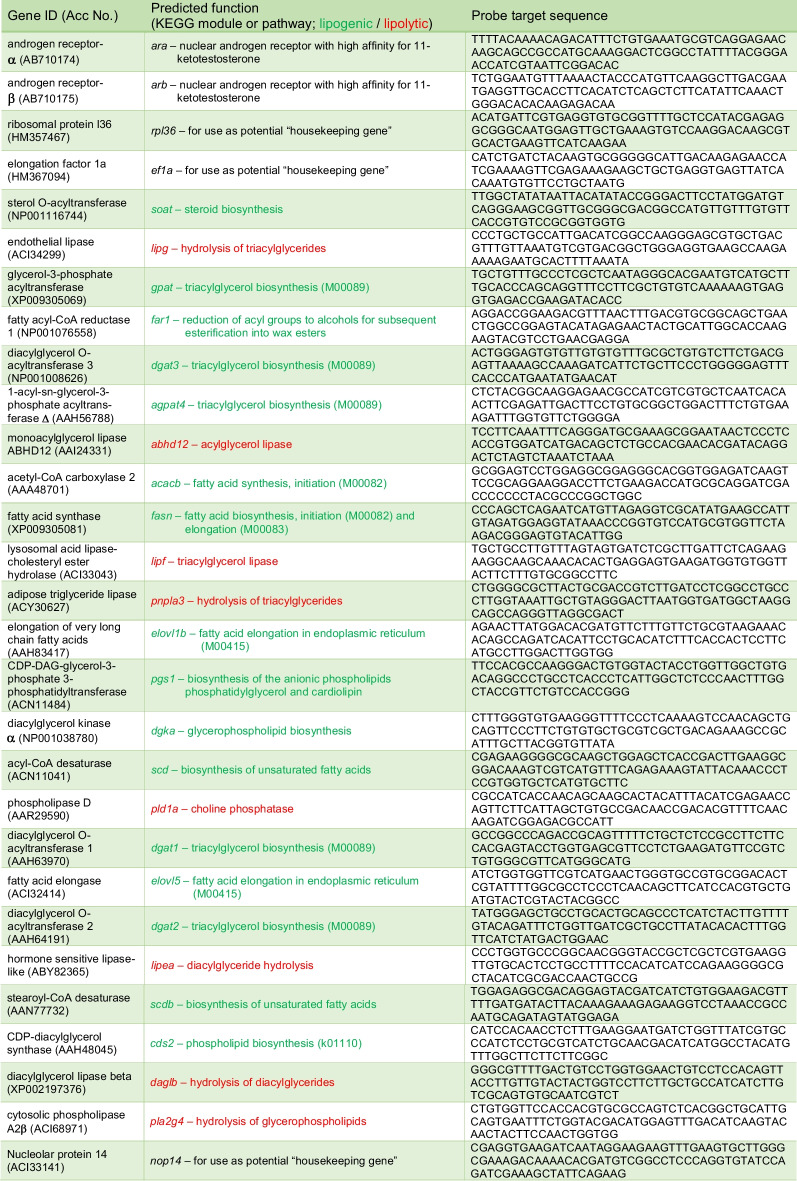
CodeSet of probe sequences coupled to specific fluorescent barcodes for quantification of white muscle target gene mRNA abundance in short-finned eel, *Anguilla australis*. Target gene sequence data were retrieved from the NCBI database or from an unpublished short-finned eel ovarian transcriptome (Unigenes)

### Reference gene selection

Stability of reference genes was assessed using BestKeeper in Excel (https://www.gene-quantification.de/bestkeeper.html) after converting gene expression quantity or geometric mean quantity (NanoString output) to a *C*_*q*_ value. A *C*_*q*_ equating to one copy was arbitrarily set at 35 (see Radke et al. 2014) and the *C*_*q*_ for reference gene mRNA levels then estimated using *C*_*q*_ = 35 – ^2^log(copy number); *C*_*q*_ values are essentially log-transformed data and thus, are likely to be normally distributed (Pfaffl et al. 2004), allowing BestKeeper to be employed for NanoString data. Reference gene expression data were then statistically analysed for stage or treatment effect using univariate general linear models (Uni-GLM).

Accordingly, NanoString-derived reference gene copy numbers from the field survey, after conversion to *C*_*q*_ and evaluation by BestKeeper, yielded three (combinations of) genes (*ef1a* + *nop14*, *ef1a* + *rpl36* and *ef1a* + *nop14* + *rpl36*) as essentially equally stable, standard deviations being lowest (*std dev* ± *CP* = 0.14–0.15 and *std dev* ± *x-fold* = 1.10–1.11) and correlations between expression of different genes > 0.8 for all comparisons.

Using the same approach for samples from yellow eels exposed to different amounts of 11KT (‘Experiment I’), then *nop14*, or combinations of genes containing *nop14*, proved most stable, but stability was also high for other genes or gene combinations tested (*std dev* ± *CP* < 0.25 and *std dev* ± *x-fold* < 1.20). However, the correlation between expression of *nop14* and that of the other two housekeeping genes was negative or strongly negative, and *nop14* was therefore removed; *ef1a* + *rpl36* was accordingly deemed the best housekeeper, and its expression was not different between the treatment groups (*F*_3,8_ = 0.836; *P* = 0.511).

Lastly, when applying BestKeeper to gene expression data from silver eels that did, or did not, receive 11KT (Experiment II), *ef1a*, *ef1a* + *rpl36* and *ef1a* + *nop14* + *rpl36* presented with the highest stability (*std dev* ± *CP* = 0.26–0.28 and *std dev* ± *x-fold* = 1.20–1.21); data were distributed similarly for both control and 11KT-treated silver eels regardless of the reference gene used (all *P* > 0.4).

On the basis of these analyses, we selected the geometric mean of *ef1a* + *rpl36* expression as the normaliser of target gene expression data across the field survey and both experiments.

### Statistical analyses

Morphometric, histological and plasma steroid data are presented as means and standard errors. Differences between means were tested on normally distributed data (after log-transformation, as needed) using Uni-GLM in the software package SPSS24 Statistics (IBM). Where relevant, post hoc multiple statistical testing was done employing Scheffé’s method, which is relatively conservative (Ruxton and Beauchamp 2008). Differences between means were considered significant for *P* < 0.05.

Differences between life history stages (yellow vs. silver eels) or effects of 11KT treatment on gene expression in silver eel muscle were first determined using Uni-GLM for each of the normalised target genes (see ‘Reference gene selection’ for reference gene selection). If homogeneity of variances could not be achieved, even after log-transformation, then the non-parametric Mann–Whitney *U* test was used to compare target gene expression means. Gene expression data in white muscle from yellow eels implanted with 11KT were evaluated using linear regression analysis as this was deemed more biologically informative (dose-dependent association with gene expression) than Uni-GLM (possible treatment effects could be limited to one dose, rather than showing a consistent dose-dependent association).

To account for multiple comparisons (26 target genes tested concurrently), false discovery rate testing was subsequently employed. Specifically, sequential false discovery rates (FDR; *P* = 0.05) according to Benjamini and Hochberg (1995) were calculated after manually ordering the probability values for all 26 target genes from least to most likely. The ordered *P* values were compared with all *Pi* = (^*i*^/_*n*_)*0.05, ranging between *P* = (^1^/_26_)*0.05 to (^26^/_26_)*0.05 for all *i* = 1–26 (i.e., the number of target genes). Both the low-stringency Uni-GLM or regression outcomes and the much more stringent FDRs are presented in the ‘Results’ section for comparison.

#### Multi-dimensional scaling and hierarchical cluster analysis

Exploratory analysis of gene expression data from yellow and silver eel white muscle was done in SPSS24 using multidimensional scaling (MDS) with two-dimensional Euclidean distances in order to represent similarities between individual fish in a 2D plot. Plots were further analysed as hierarchical clustering analysis using Ward’s linkage and squared Euclidean distance. Subsequently, the measures obtained from eels in Experiments I and II were analysed together with the data from the field survey to explore whether 11KT treatment resulted in a ‘shift’ of gene expression data towards the silver eel phenotype. MDS and hierarchical cluster analysis was run as a descriptive analysis and animal groupings were not statistically tested.

## Results

### Field survey

#### Fish descriptives

In keeping with earlier reports (e.g. Lokman et al. 1998, 2010), silver eels presented with typical GSI values averaging 3.24 ± 0.12%, nearly eightfold higher than those of yellow eels (0.43 ± 0.09%). These observations were reflected in larger oocyte diameters (0.26 ± 0.01 mm vs. 0.12 ± 0.01 mm; *F*_1,10_ = 137.07; *P* < 0.001) and coincided with HSIs that were nearly double those (*F*_1,10_ = 45.85; *P* < 0.001) in silver (1.04 ± 0.02%) compared to those in yellow eels (0.58 ± 0.05%). Mirroring these increases in biometric measures were elevated levels of 11KT in plasma, silver eels presenting with concentrations of 41–67 ng/mL (*n* = 6) and yellow eels with 0.13–2.91 ng/mL (*n* = 6).

#### Expression of candidate genes in white muscle

**Fig. 1:**
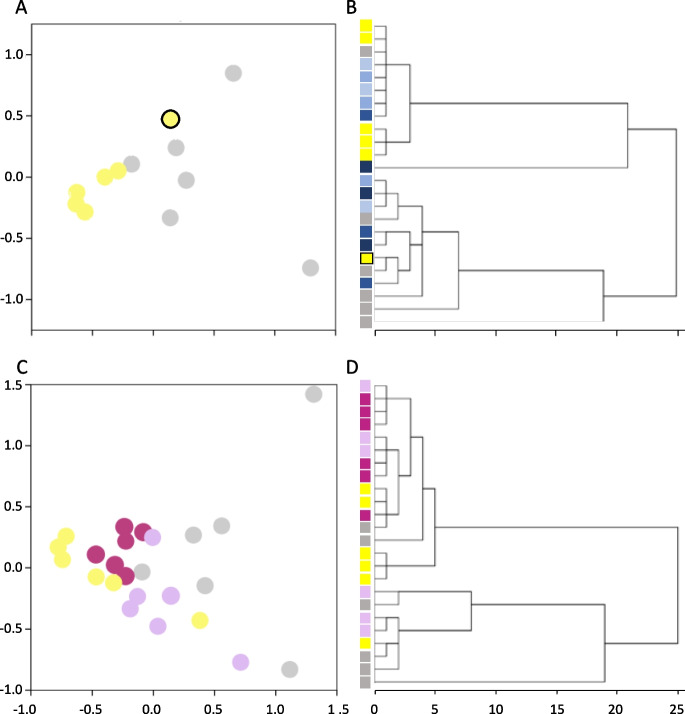
Multidimensional scaling (MDS) and hierarchical clustering (Ward’s linkage) plots based on target gene expression (26 genes; for detail, see text) in white muscle of female short-finned eel, *Anguilla australis*. **A** MDS positions of yellow (  -colored symbols) and silver eels (  -colored symbols) soon after capture from the wild (eels sampled for the “Field survey,” see Section “Experimental design” of the text); **B** clustering of yellow eels (eels from “Experiment I—In vivo 11-ketotestosterone dose response in female yellow short-finned eels”) implanted with 0, 0.03, 0.1 or 0.3 mg 11-ketotestosterone-containing pellets (  -colored symbols, increasing darkness reflecting increasing dose) alongside yellow (  ) and silver eels (  ) from the wild (eels from the ‘Field survey’)—note the weak association between yellow eels and control/low dose implants and that between silver and medium/high dose implants; **C** and **D**) MDS positions and hierarchical clustering of captive silver eels (eels from ‘Experiment II—In vivo 11-ketotestosterone treatment of female silver short-finned eels’) implanted with 0 (  -colored symbols) or 0.3 mg pellets (  -colored symbols) containing 11-ketotestosterone alongside yellow and silver eels from the wild (eels from the ‘Field survey’). The *X*- and *Y*-axes represent distance (arbitrary scale)

Using white muscle Nanostring™ gene expression data, MDS resulted in strong clustering of five among six yellow eels (Fig. 1A), but silver eels were more dissimilar to each other. In addition, some overlap between yellow and silver eels was evident in the 2D space that described proximities. MDS plots were reflected in cluster analysis, which yielded two major groups of six animals each (data not shown): one cluster with five yellow eels and one silver eel, and a second, reverse-composition cluster. The generic separation of the eels into two clusters by stage was reflected in statistical evaluation of the data; thus, mRNA levels of thirteen of the 26 target genes expressed in white muscle were significantly higher in silver than in yellow eels (Table 2), both when statistically analysed as stand-alone contrasts for individual genes and when applying a sequential FDR correction at *α* = 0.05. Lipases were over-represented in the list of differentially expressed genes, especially when considering the barely detectable levels of hormone-sensitive lipase (< 10 copies/sample). Unexpectedly, the most highly expressed gene, in terms of absolute copy numbers, was *ara* (10,245 ± 1838 copies/100 ng RNA) in white muscle of silver eels, but expression was not different (*F*_1,10_ = 3.295; *P* = 0.100) to that of yellow eels (7411 ± 719 copies/100 ng RNA) after implementing normalisation over the *ef1a* + *rpl36* geometric mean; other highly expressed genes were *acetyl-CoA carboxylase* (2357–7889 copies/100 ng RNA) and *glycerol-3-phosphate acyltransferase* (4503 ± 1905 copies/100 ng RNA), both considered as having lipogenic activity. The most abundantly expressed ‘lipolytic genes’ in silver eel white muscle (/100 ng total RNA) were *diacylglycerol lipase β* (1100–1821 copies), *monoacylglycerol lipase ABHD12* (345–765 copies) and *lysosomal acid lipase-cholesteryl ester hydrolase* (552–671 copies), all of which were significantly upregulated in this study (Table 2).

**Table 2:**
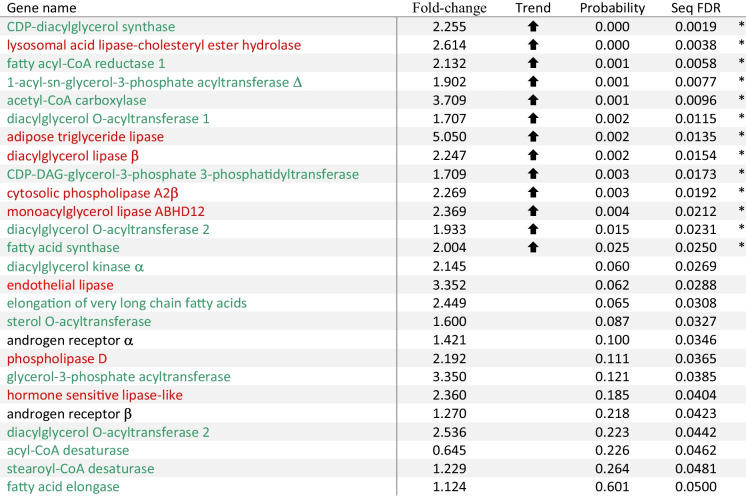
Differences in expression (normalised over geometric mean of elongation factor1α and ribosomal protein l36 expression) of genes encoding the nuclear androgen receptors or enzymes with lipolytic or lipogenic activities in white muscle between wild-caught yellow and silver female eel, *Anguilla australis* using NanoString analysis

Fold-change represents the ratio silver/yellow gene expression ratio. ⬆ and ⬇ signify the direction of a difference in expression of the target gene in silver compared to yellow eels. Differences between means (*n* = 6) were determined using one-way univariate ANOVA or Mann-Whitney *U* test (‘probability’) and false discovery rate corrections (‘seq FDR’; significant for genes identified with *) were made according to Benjamini and Hochberg (1995)

### Experiment I—In vivo 11-ketotestosterone dose response in female yellow eels

#### Fish descriptives

**Fig. 2:**
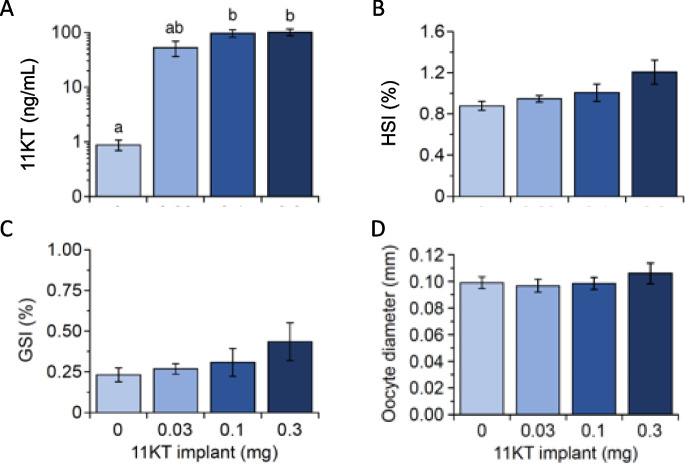
Effects of administration of slow-release implants containing 0, 0.03, 0.1 or 0.3 mg 11-ketotestosterone (11KT) for 3 weeks on **A** plasma levels of 11KT, **B** hepatosomatic index (HSI), **C** gonadosomatic index (GSI) and **D** oocyte diameter in yellow short-finned eel, *Anguilla australis*. Data represent the means ± SEM of 5–6 fish. Letters above the bars indicate significant differences between treatment groups

One of the females in the M0.1 group died from unknown causes during the experiment. Remaining animals were in good condition throughout the experimental period. 11KT treatment effectively elevated plasma 11KT levels in a clear dose-dependent fashion, mean values increasing from 0.9 ng/mL in control fish to 52, 96 and 101 ng/mL in the L0.03, M0.1 and H0.3 groups (all *n* = 5–6), respectively (see Fig. 2A). 11KT levels in the subsets of fish (*n* = 3) randomly selected for the NanoString analysis were largely comparable to the group means, averaging 0.7, 60, 93 and 125 ng/mL with increasing 11KT implant dose from 0 to 0.3 mg. Experimental increases in 11KT levels were reflected in near-significant main effects on HSI (*F*_3,20_ = 2.258, *P* = 0.113; Fig. 2B) and GSI (*F*_3,20_ = 3.010, *P* = 0.054; Fig. 2C), but less so on oocyte diameter (*F*_3,20_ = 0.561, *P* = 0.647; Fig. 2D); moreover, when defining dose as a continuous, rather than as a fixed variable, significant associations between implant dose and HSI (*R*^2^ = 0.19; *P* = 0.033) and GSI (*R*^2^ = 0.29; *P* = 0.007) were found (see Fig. 2B and C). Two of the fish in the H0.3 group stood out as having black pectoral fins, which is an androgen-dependent phenotypic presentation (Lokman et al. 1998), and one of these fish was included in the NanoString-determined gene expression.

#### Expression of candidate genes in white muscle of 11KT-treated yellow eels

MDS of gene expression data from fish in Experiment I yielded a small cloud of subjects that contained all fish belonging to the control and L0.03 groups, together with one fish from the M0.1 and one from the H0.3 group; the remaining four individuals were more dissimilar to the cluster, an observation that was especially noticeable for one female in the H0.3 group with black pectoral fins (data not shown). Using hierarchical clustering, the outlier in H0.3 was separate from the three fish that were outside the MDS cloud, and the remaining eight females were split across two small clusters without clear association with 11KT treatment regime (data not shown). When including yellow and silver eels in the exploratory MDS, the general topography was retained, four fish (two each from the M0.1 and H0.3 groups) being noticeably separated from a cluster with mostly yellow eels and control or L0.03 fish; three of these four fish occupied the generic space where silver eels were found. The fourth individual (H0.3 with black pectoral fins) was highly dissimilar in terms of white muscle target gene expression to all other fish in the analysis (data not shown). These observations were reflected in hierarchical cluster analysis, in which two individuals did not cluster (one silver eel and one fish in the H0.3 group); the remaining fish were split into two main groups: one group representing most of the yellow eels and most of the low-dose treatment groups, the other representing most silver eels and most of the high-dose treatment groups (see Fig. 1B).

**Fig. 3:**
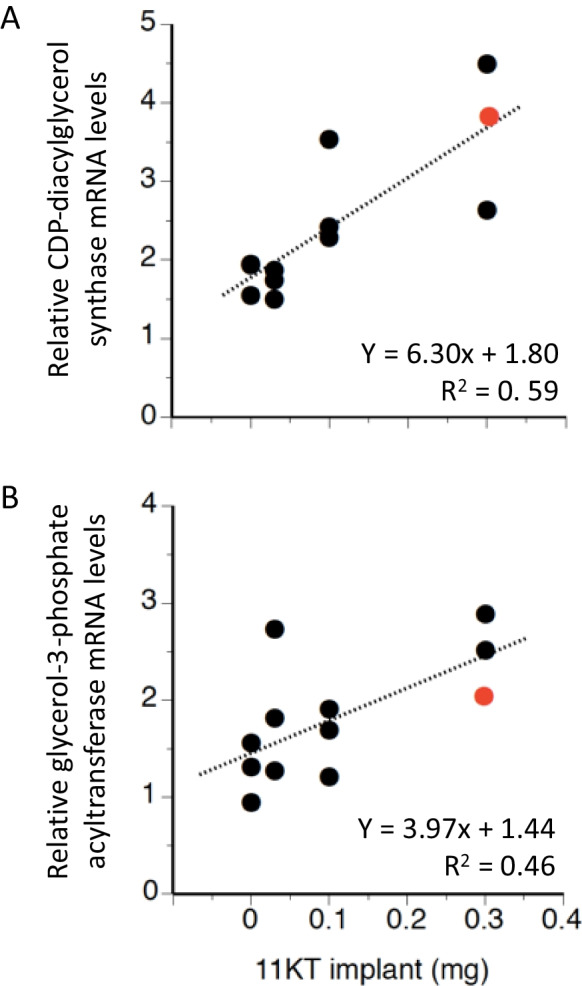
Relationship between administered levels of 11-ketotestosterone (11KT) in slow-release implants (0–0.3 mg/fish) and expression of CDP-diacylglycerol synthase (**A**) and glycerol-3-phosphate acyltransferase (**B**) in white muscle of female yellow short-finned eel, *Anguilla australis*. Statistical analyses were conducted on data from 11 eels; data from a 12th female (red symbol) were excluded as this individual was a clear outlier in exploratory data analysis of the entire dataset (see text and Fig. 1B). Inclusion of this 12^th^ fish only marginally changed the slope of the regression line and the correlation coefficient for both plots (not shown)

Interrogation of gene expression data confirmed the presence of a clear outlier which presented with mRNA levels that were, for several target genes, an order of magnitude higher than that of all other fish. This eel, already described above as belonging to the H0.3 group, was therefore omitted from analysis. The remaining 11 fish were subjected to regression analysis, which identified two genes for which expression positively correlated with 11KT dose, i.e., *CDP-diacylglycerol synthase* (Fig. 3A) and *glycerol-3-phosphate acyltransferase* (Fig. 3B). For a third gene, *diacylglycerol lipase β*, a positive trend (*P* = 0.086) was evident (Table 3). Interestingly, for each of these three genes, mRNA levels in the omitted fish were similar to those of other fish in the H0.3 treatment group. When imposing Benjamini and Hochberg’s sequential FDR, no significant associations were found among the 26 target genes.

**Table 3:**
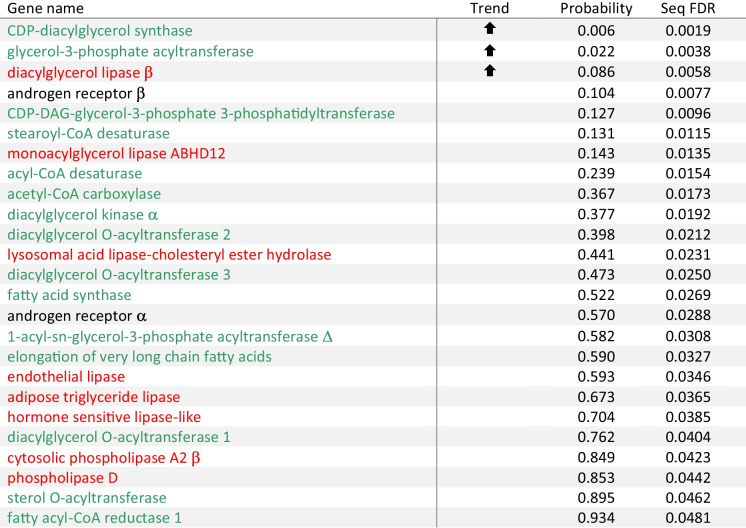
Correlation between treatment dose of 11-ketotesterosterone (11KT) and expression (normalised over geometric mean of elongation factor1*α* and ribosomal protein l36 expression) of genes encoding the nuclear androgen receptors or enzymes with lipolytic or lipogenic activities in white muscle of yellow female eel, *Anguilla australis* using Nanostring analysis

Associations were tested using linear regression analysis (‘probability’) and false discovery rate corrections (‘seq FDR’; significant for genes identified with *) were made according to Benjamini and Hochberg (1995). ⬆ and ⬇ signify whether associations between 11KT implant dose (0, 0.03, 0.1, 0.03 mg/fish; total *n* = 11 fish) and target gene expression are positive or negative

### Experiment II: In vivo 11-ketotestosterone treatment of 11KT-silver female eels

#### Fish descriptives

**Fig. 4:**
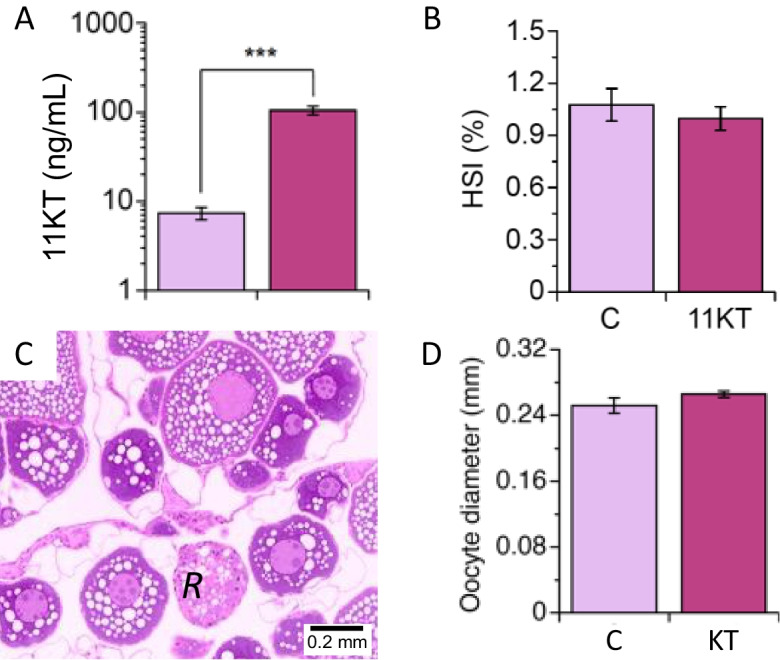
Effects of administration of slow-release implants containing 0 (**C**) or 0.3 mg 11-ketotestosterone (11KT) for 3 weeks on **A** plasma levels of 11KT, **B** hepatosomatic index (HSI), **C** ovarian histology and evidence of atresia (*R*) and oocyte diameter **D** in silver short-finned eel, *Anguilla australis* after 7 months of captivity. Data represent the means ± SEM of six fish. Triple asterisks (***) indicate a significant difference between treatments at *P* < 0.001

11KT implants effectively increased levels of the androgen in plasma to 105 ± 11.6 ng/mL, well over tenfold the levels (7.3 ± 1.2 ng/mL) seen in control silver eels held captive for around 7 months (Fig. 4A). Increased levels of 11KT were reflected in increased GSI (control group: 2.14 ± 0.14%; 11KT-treated group: 2.60 ± 0.21%), but less so in oocyte diameter (0.25 ± 0.01 mm vs. 0.27 ± 0.00 μm; Fig. 4D). Follicular atresia was widespread, but not specifically quantified, in ovaries from eels in both treatment groups (Fig. 4C). HSI was not affected, averaging close to 1.0% in both groups (Fig. 4B).

#### Expression of candidate genes in white muscle from 11KT-treated silver eels

Gene expression differences between blank and 11KT-implanted silver eels were visualised in the MDS plot and hierarchical clustering analysis, in which especially the androgen-treated fish clustered tightly (data not shown). When including wild-caught yellow and silver eels in the analysis, tight clustering of 11KT-treated silver eels was retained (burgundy symbols in Fig. 1C), and the space they occupied was in between that of the yellow and silver females. Blank-implanted controls clustered notably less in a space that was also crudely halfway between yellow and silver females in dimension 1, but less so in dimension 2 (Fig. 1C). Ward’s linkage yielded weak evidence for clustering, captive control and wild silver eels tending to group in one cluster, and yellow eels and 11KT-treated silver eels in another cluster. However, despite a bias in composition of both clusters, the lack of a firm separation was clearly noticeable, as shown in Fig. 1D.

Surprisingly, despite notable segregation of blank- and 11KT-implanted silver females by MDS, target gene expression in muscle changed little between both groups; only relative *ara* expression was different between both experimental groups, and although highly significant (*F*_1,10_ = 13.098, *P* = 0.005), fold-change was modest (*ara* mRNA levels in 11KT-treated eels were 74% those of controls). Interestingly, a strong differing trend was also detected for *arb* (*F*_1,10_ = 3.87, *P* = 0.077), but in the opposite direction (1.38-fold change). Similarly, expression of the lipolytic enzyme *lysosomal acid lipase-cholesteryl ester hydrolase* was near-significantly up-regulated (1.32-fold; *F*_1,10_ = 4.685, *P* = 0.056) in 11KT-treated females. Remaining genes were expressed at very similar levels in both treatment groups, fold-change mostly (17 among 23 genes) ranging between 0.8 and 1.2 (Table 4). The small number of (near-)significant contrasts did not reach the 5% threshold when imposing an FDR correction (Table 4).

**Table 4:**
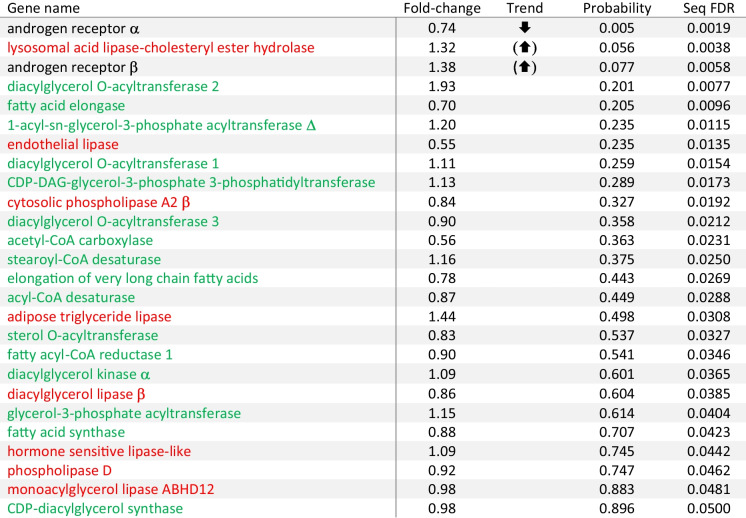
Expression (normalised over geometric mean of elongation factor 1*a* and ribosomal protein l36 expression) of genes encoding the nuclear androgen receptors or enzymes with lipolytic or lipogenic activities in white muscle of female short-finned eel, *Anguilla australis*

Starved silver eels received sustained-release implants of 11-ketotestosterone at 0 or 0.3 mg per fish for three weeks (*n* = 6 fish/group). Gene expression was analysed using Nanostring Technologies. ⬆ and ⬇ signify an increase and decrease, respectively, in expression of the target gene in 11KT-treated fish compared to controls using univariate general linear models (‘probability’). Corrections for multiple comparisons were made by adopting false discovery rates (‘seq FDR’; significant for genes identified with *) according to Benjamini and Hochberg (1995)

## Discussion

Yellow eels are in the non-migratory phase and are characterised by feeding and somatic growth. Silver eels, on the other hand, have ceased feeding and are pre-adapting/pre-adapted to an oceanic migratory existence that requires endogenous fuel to support sustained swimming and oocyte development. As many of the changes seen during silvering are inducible by exogenous 11KT treatment, and 11KT has previously been implicated in several aspects of reproduction-related lipid physiology, it seemed plausible that 11KT could act as a master co-ordinator for all lipid physiology-related changes. Specifically, we sought to answer the question whether 11KT could be the hormonal driver of lipid mobilisation from white muscle lipid depots by changing the expression of lipid storage-related genes.

Our first approach to testing our hypothesis was to do a ‘stock take’ and compare the expression of 26 candidate genes in white muscle between yellow and silver eels. The fish used for this study were captured in a coastal brackish lake (c.f., Arai et al. 2004) and therefore, were probably pre-adapted (increased red muscle mass; see Pankhurst 1982), but not exercised quite yet. Sampled eels in our study can be considered representative of the yellow and silver ontogenetic stages on the basis of previous observations made on biometry and plasma steroids in short-finned eels (e.g., Lokman et al. 1998, 2010; Damsteegt et al. 2015b). Surprisingly, however, at the level of gene expression in white muscle, the clustering of yellow and silver eels was not clear-cut, one individual of either group associating with the ‘wrong’ phenotype cluster. The reason for this observation is not clear; conceivably, white muscle gene expression profiles may be somewhat confounded by stress-associated mobilisation of triacylglycerides (c.f. da Santa Lopes et al. 2023) during the lag from capture to terminal sampling later that day; this explanation is reinforced by the notion that the last fish (a yellow eel) that was sampled in the field was the outlier in the clustering analysis (Fig. 1A). In addition, the interval between terminal sampling and the last intake of food will have differed between individuals.

As expected, the expression of many of the candidate genes nonetheless differed in white muscle of eels between both life history stages. Thus, expression of five of the candidate genes encoding enzymes associated with hydrolysis of acylglycerides were upregulated in white muscle of silver females, and that of a sixth gene (*endothelial lipase*) was near-significantly increased (*P* = 0.062); only mRNA levels of the genes encoding *phospholipase D* and *hormone-sensitive lipase* (*hsl*) were not significantly upregulated, but the expression of both enzymes was at least an order of magnitude lower than that of most other lipase-like enzymes and the resulting mRNA abundance estimates may not be biologically relevant. These findings therefore suggest that Hsl is not important for lipolysis of acylglycerides in white muscle, at least not while silver eels are still inshore, thus contradicting the mechanistic assumptions about Hsl made by Van Ginneken et al. (2018). However, notwithstanding the predicted upregulation of lipolytic enzymes in white muscle of silver eels, many of the ‘lipogenic’ enzyme-encoding genes (including *acetyl-CoA carboxylase* and *fatty acid synthase*, *fasn*, both of which are associated with *de novo* lipogenesis, DNL; see Saponaro et al. 2015) were also upregulated. Interestingly, a link between lipolysis and lipogenesis, albeit considered opposing pathways, was also reported in mice chronically treated with the β3-adrenergic agonist CL 316,243 (CL; Mottillo et al. 2014). Thus, in the first few days of treatment with CL, a rapid decline in body fat was evident in these mice, but the decline stabilised thereafter—this was associated with generically increased lipid turn-over, mediated by adipose triglyceride lipase and reflected in increased expression of genes and proteins involved in both lipid catabolism and DNL (e.g., *fasn*, and glycerol kinase; see Mottillo et al. 2014). Lipolytic and lipogenic responses were also same-directional in endurance-trained rats, both being reduced compared to observations on sedentary controls (Pistor et al. 2015). Furthermore, same-directional responses for both processes were observed in rat adipocytes in response to short-chain fatty acids (Heimann et al. 2015). Other studies, again on rat adipocytes, have indicated that lipolysis can be stimulated to yield free fatty acids (FFA) to a level well beyond what is required to meet whole-body energy demand, even during fasting; as a result, some 30% of these FFA may be re-esterified (i.e., lipogenesis) into triacylglycerides (Reshef et al., 2003), providing an intracellular mechanism (as opposed to hormone-mediate extra-cellular mechanism) that can contribute to controlling adipocyte FFA levels (Li et al., 2022). Notwithstanding the added complexities of lipid homeostasis in association with thermogenesis in endothermic mammals (see Grabek and Sprenger 2024; Keipert et al. 2024), our observations on concurring increases in expression of lipolytic and (*de novo*?) lipogenic enzymes, thus, could be indicative of increased lipid cycling in white muscle of silver eels (c.f., Mottillo et al. 2014).

Are the observed differences in gene expression profiles between yellow and silver eels attributable to exposure to the androgen 11KT? To address this question, yellow eels were implanted with different amounts of 11KT, and clear trends for increased GSI and HSI were observed. Furthermore, eels in the control and L0.03 treatment groups weakly clustered with wild-caught yellow eels, and the fish in the M0.1 and H0.3 groups with the silver eels. The experimental paradigm was therefore considered effective, but how was this reflected in target gene expression? When employing regression analysis—which we deemed the tidiest approach to detect a dose–response effect—the expression of only two genes, both lipogenic, were upregulated: that encoding *CDP-diacylglycerol synthase* and that for *glycerol-3-phosphate acyltransferase*. These findings are notably different from the observations on white muscle from yellow and silver eels, suggesting that 11KT is not effective at mimicking the silver eel gene expression profile.

Following implantation of starved, captive silver eels—associated with reduced GSI and clear evidence of ovarian atresia—with 11KT, no support for clustering of wild-caught silver eels and 11KT-treated captive silver eels could be found. Moreover, 11KT only brought about a reduction in expression of *ara*, and a tendency for an increase in expression of *arb* and of the lipolytic enzyme-encoding gene *lysosomal acid lipase-cholesteryl ester hydrolase* in white muscle of 11KT-treated silver eels. These observations indicate that androgens presumably affect muscle physiology, reinforced by the high expression of *ar* genes in this tissue, but that androgen action in long-held captive silver eels does not result in a white muscle gene expression profile that is reminiscent of that in wild-caught silver eels. We therefore do not find support for our hypothesis that 11KT induces the target gene expression profile in white muscle as seen in the silver eel. However, we acknowledge that the prolonged period of starvation prior to experimentation conceivably resulted in a physiological state different from that of a ‘fresh’ migrant, and hence, that 11KT action in this experiment may not mirror that at the onset of migration.

Are there other factors that might mediate acylglyceride lipolysis from white muscle stores immediately prior to migration? A role for AMP-activated protein kinase (AMPK) was highlighted in a review by Magnoni et al. (2013) that evaluated the control of fuel utilization in muscle—the activity of this enzyme is governed by the ratio of AMP to ATP, and hence, a reflection of the ‘energy status’ of the muscle. However, AMPK may not directly be involved in regulating lipolysis from intramuscular stores (presumably adipocytes; see Sheridan 1994; Grabner et al. 2021), prompting the search for other mediators. Could cortisol play this role, given its previously demonstrated lipolytic actions in the European eel, albeit in response to an experimentally stressful paradigm (Lidman et al. 1979)? Cortisol has been considered by some authors (e.g., Van Ginneken et al. 2007; Aedo et al. 2021; Pfalzgraff et al. 2021) as the regulator of fuel mobilisation, but evidence in support of such a role in anguillids is not compelling, if not altogether absent; rather, the proposed role of cortisol as stimulator of lipolysis presumably largely hinges on its ‘catabolic nature’ (c.f. Peckett et al. 2011) and on the extrapolation of the Pacific salmon ‘scenario’. Indeed, Van Ginneken et al. (2007) assigned a possible role for cortisol in mobilisation of fuels merely on the basis of seasonally changing blood levels of this glucocorticoid, concentrations proving higher in silver than yellow European eels subjected to capture and handling stress. These observations squarely contrast those on *A. australis*, in which handling-stressed yellow eels presented with higher cortisol levels than silver eels (Lokman et al. 1998; Chia 2017). Moreover, near-basal levels of < 10 ng/mL in the latter species at the onset of migration (Lokman et al. 1998) are hardly suggestive of a state of hypercortisolemia that is seen in Pacific salmon (*Oncorhynchus* spp.), as reported, for example, by McBride and co-workers (1986). Furthermore, the documented increase in cortisol levels in pink salmon appears to occur towards the end of migration (c.f. McBride et al. 1986), at a time when lipids stores, at least in pink salmon, have been largely exhausted and protein catabolism appears to be initiated (see McBride et al. 1986; Morash et al. 2013). Indeed, the absence of a dramatic increase in cortisol levels in most eels (five among eight) subjected to forced swimming for 6 weeks (Palstra et al. 2009) casts further doubt onto its role as principal lipid mobiliser. Lastly, and notwithstanding the notable differences in life histories and locations of lipid depots between different species of fish (see Sheridan 1994), the apparent absence of differentially expressed genes encoding lipolytic enzymes, but not those implicated in protein catabolism, in exercised zebrafish (Palstra et al. 2019), further hints at a limited effect of cortisol on lipid mobilisation. Indeed, there is evidence to suggest that cortisol can even have lipogenic effects in humans, especially under situations of chronic stress (Peckett et al. 2011).

In summary, there is a clear difference in the white muscle gene expression profile between yellow and silver short-finned eels—a substantial number of genes encoding enzymes belonging to both the lipolytic and (*de novo*?) lipogenic pathways were differentially expressed. Treatment of yellow eels and of long-term captive silver eels with sustained-release 11KT implants effected some change in the gene expression profile between groups, but the resulting profiles did not resemble those of wild-caught silver eels. We therefore do not support the prediction that 11KT is responsible for the different profiles in white muscle between yellow and silver eels. Additional experimentation, possibly involving prolonged swimming/simulated migration, is required to understand how the fuelling of lipid mobilisation during the spawning migration of anguillid eels is regulated.

## Data Availability

The data presented in this paper are curated by the corresponding author. Raw data will be provided on reasonable request to the corresponding author.
